# Acute Response in the Noninfarcted Myocardium Predicts Long-Term Major Adverse Cardiac Events After STEMI

**DOI:** 10.1016/j.jcmg.2022.09.015

**Published:** 2023-01

**Authors:** Mayooran Shanmuganathan, Ambra Masi, Matthew K. Burrage, Rafail A. Kotronias, Alessandra Borlotti, Roberto Scarsini, Abhirup Banerjee, Dimitrios Terentes-Printzios, Qiang Zhang, Evan Hann, Elizabeth Tunnicliffe, Andrew Lucking, Jeremy Langrish, Rajesh Kharbanda, Giovanni Luigi De Maria, Adrian P. Banning, Robin P. Choudhury, Keith M. Channon, Stefan K. Piechnik, Vanessa M. Ferreira, OxAMI Study Investigators

**Affiliations:** a Acute Vascular Imaging Centre (AVIC), University of Oxford, John Radcliffe Hospital, Oxford, United Kingdom; b Oxford Centre for Clinical Magnetic Resonance Research (OCMR), John Radcliffe Hospital, National Institute for Health Research (NIHR) Oxford Biomedical Research Centre, Oxford BHF Centre of Research Excellence, University of Oxford, Oxford, United Kingdom; c Oxford University Hospitals NHS Trust, John Radcliffe Hospital, Oxford, United Kingdom

**Keywords:** CMR, heart failure, MACE, myocardial injury, noninfarcted myocardium, prognosis, STEMI, T1-mapping, AAR, area at risk, CMR, cardiac magnetic resonance, IDI, Integrated Discrimination Index, IMH, intramyocardial hemorrhage, IS, infarct size, LGE, late gadolinium enhancement, LVEF, left ventricular ejection fraction, MACE, major adverse cardiac events, MI, myocardial infarction, MVO, microvascular obstruction, NRI, Net-Reclassification Index, NT-proBNP, N-terminal pro–B-type natriuretic peptide, PPCI, primary percutaneous coronary intervention, STEMI, ST-segment elevation myocardial infarction

## Abstract

**Background:**

Acute ST-segment elevation myocardial infarction (STEMI) has effects on the myocardium beyond the immediate infarcted territory. However, pathophysiologic changes in the noninfarcted myocardium and their prognostic implications remain unclear.

**Objectives:**

The purpose of this study was to evaluate the long-term prognostic value of acute changes in both infarcted and noninfarcted myocardium post-STEMI.

**Methods:**

Patients with acute STEMI undergoing primary percutaneous coronary intervention underwent evaluation with blood biomarkers and cardiac magnetic resonance (CMR) at 2 days and 6 months, with long-term follow-up for major adverse cardiac events (MACE). A comprehensive CMR protocol included cine, T2-weighted, T2∗, T1-mapping, and late gadolinium enhancement (LGE) imaging. Areas without LGE were defined as noninfarcted myocardium. MACE was a composite of cardiac death, sustained ventricular arrhythmia, and new-onset heart failure.

**Results:**

Twenty-two of 219 patients (10%) experienced an MACE at a median of 4 years (IQR: 2.5-6.0 years); 152 patients returned for the 6-month visit. High T1 (>1250 ms) in the noninfarcted myocardium was associated with lower left ventricular ejection fraction (LVEF) (51% ± 8% vs 55% ± 9%; *P =* 0.002) and higher NT-pro-BNP levels (290 pg/L [IQR: 103-523 pg/L] vs 170 pg/L [IQR: 61-312 pg/L]; *P =* 0.008) at 6 months and a 2.5-fold (IQR: 1.03-6.20) increased risk of MACE (2.53 [IQR: 1.03-6.22]), compared with patients with normal T1 in the noninfarcted myocardium (*P =* 0.042). A lower T1 (<1,300 ms) in the infarcted myocardium was associated with increased MACE (3.11 [IQR: 1.19-8.13]; *P =* 0.020). Both noninfarct and infarct T1 were independent predictors of MACE (both *P =* 0.001) and significantly improved risk prediction beyond LVEF, infarct size, and microvascular obstruction (C-statistic: 0.67 ± 0.07 vs 0.76 ± 0.06, net-reclassification index: 40% [IQR: 12%-64%]; *P =* 0.007).

**Conclusions:**

The acute responses post-STEMI in both infarcted and noninfarcted myocardium are independent incremental predictors of long-term MACE. These insights may provide new opportunities for treatment and risk stratification in STEMI.

Survival rate after acute ST-segment elevation myocardial infarction (STEMI) has dramatically improved in recent times through primary percutaneous coronary intervention (PPCI) and optimal medical therapy.^1^ However, some patients experience poor long-term cardiovascular outcomes, such as heart failure, arrhythmias, and cardiac death.^1^ Thus, early identification of predictors of such adverse outcomes is desirable to improve long-term prognosis after STEMI.

The degree of acute injury to the infarcted myocardium, such as infarct size (IS), or the presence of microvascular obstruction (MVO), and intramyocardial hemorrhage (IMH), is a known independent predictor of both short- and long-term clinical outcomes post-STEMI.^2^ The noninfarcted myocardium includes the salvaged area at risk (AAR) and the remote zone farthest away from the infarction (Central Illustration). Emerging translational and clinical evidence suggests that the noninfarcted remote myocardium may also exhibit acute inflammation and injury post myocardial infarction (MI).^3^, ^4^, ^5^, ^6^, ^7^ These acute pathophysiologic changes are thought to be mediated by the innate immune response^3^^,^^4^ and may lead to maladaptive matrix changes, resulting in adverse left ventricular (LV) remodeling and poor long-term outcomes.^7^, ^8^, ^9^ Characterization of the noninfarcted myocardium immediately post-STEMI may therefore help to improve risk stratification and identify therapeutic targets for cardio-protection against heart failure.

**Central Illustration:**
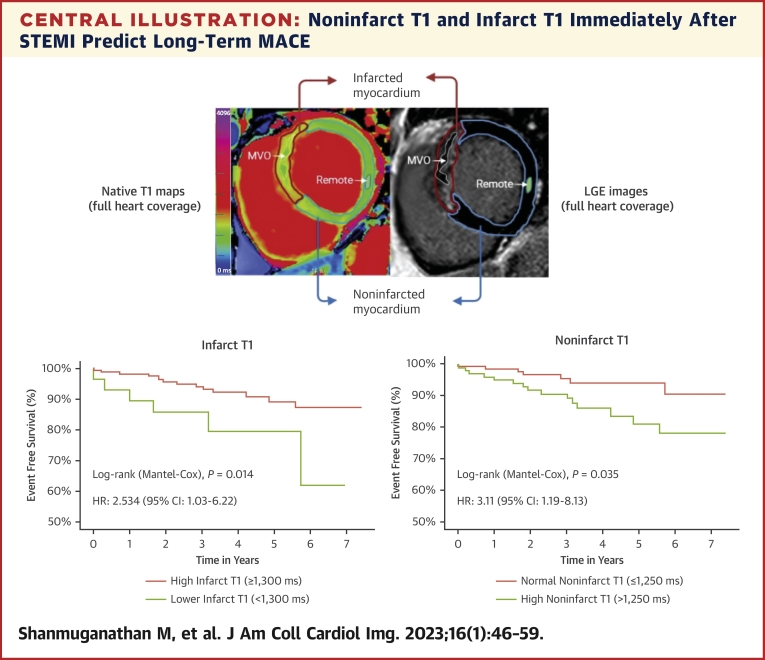
Noninfarct T1 and Infarct T1 Immediately After STEMI Predict Long-Term MACE Current assessment of myocardial tissue in patients with ST-segment elevation myocardial infarction (STEMI) focuses on the infarcted myocardium, which is the area with late gadolinium enhancement (LGE) on cardiac magnetic resonance (CMR). This study describes, for the first time, the prognostic significance of myocardial injury in the entire heart using CMR T1-mapping, in both infarcted and noninfarcted myocardium (area without LGE, which includes the remote myocardium). A “lower” infarct T1 (≤1,300 ms, reflecting the presence of microvascular injury such as MVO) and a high noninfarct T1 (>1,250 ms) measured at 2 days after STEMI are associated with long-term MACE (new diagnosis of symptomatic heart failure or sustained ventricular arrhythmia or cardiac death). MACE = major adverse cardiac events; MVO = microvascular obstruction.

Multiparametric cardiac magnetic resonance (CMR) imaging is a powerful noninvasive tool to interrogate myocardial tissue after STEMI.^2^ Advanced CMR techniques, such as T1-mapping, are highly sensitive for detecting increased free water content in acute myocardial injury, particularly MI.^10^, ^11^, ^12^ Elevated T1 values can delineate the edematous AAR^13^ and predict the final IS post-STEMI.^14^ Lowered T1 in the core of the infarct, thought to reflect MVO and/or IMH, is inversely associated with short-term negative remodeling of the LV and long-term adverse outcomes.^15^ In the noninfarcted remote myocardium, elevated T1 is associated with negative remodeling of the LV and adverse outcomes in patients at 6 months after STEMI.^6^^,^^7^ Thus, changes in the noninfarcted myocardium may also carry important clinical significance; however, their long-term prognostic relevance is unclear.

In this study, we hypothesized that, in addition to changes in the infarcted myocardium, the noninfarcted myocardium may also demonstrate various grades of acute response immediately post-STEMI. We sought to detect these changes noninvasively using CMR and to determine whether these changes may predict long-term major adverse cardiac events (MACE).

## Methods

### Study population and treatment

**Figure 1:**
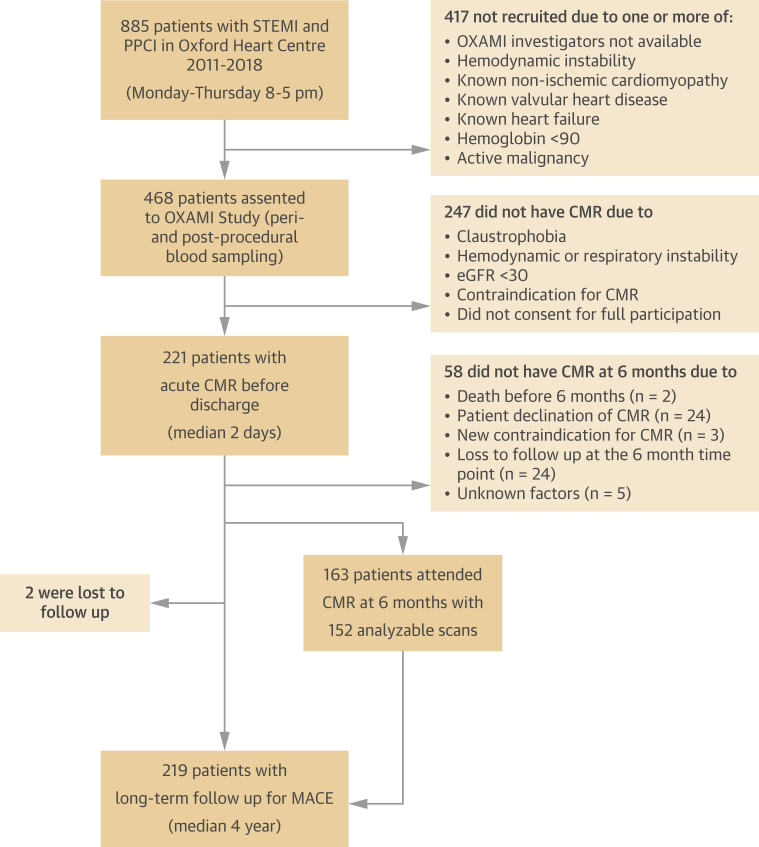
Study Flowchart Patients with STEMI were prospectively recruited between 2011 and 2018. CMR = cardiac magnetic resonance; eGFR = estimated glomerular filtration rate; MACE = major adverse cardiac events; OXAMI = Oxford Acute Myocardial Infarction; PPCI = primary percutaneous coronary intervention; STEMI = ST-segment elevation myocardial infarction.

Patients with STEMI admitted to our center for PPCI were prospectively enrolled in the OxAMI (Oxford Acute Myocardial Infarction) study (Figure 1).^16^^,^^17^ The study protocol was approved by the local ethics committee (REC:10/H0408/24). All participants provided written informed consent. Patients underwent blood sampling at regular intervals during the hospital stay and CMR typically before hospital discharge. They were invited for a follow-up CMR scan and blood sampling at 6 months post-discharge.

Patients were eligible if the onset of symptoms had been <12 hours before PPCI and if they had ST-segment elevation of at least 0.1 mV in >2 contiguous limb leads or at least 0.2 mV in >2 contiguous precordial leads. Standard exclusion criteria included severe heart valve disease, known cardiomyopathy, hemodynamic instability lasting >12 hours after revascularization, contraindications to CMR, and non-MI diagnoses of ST-segment elevation syndromes (eg, takotsubo cardiomyopathy and myocarditis). PPCI strategy and treatment decisions were left to the operator’s discretion.

### CMR imaging

Scans were performed on 3.0-T CMR scanners (either MAGNETOM Tim Trio or MAGNETOM Verio, Siemens Healthcare).^14^ Briefly, the CMR protocol included cine, native T1-mapping (Shortened Modified Look-Locker Inversion recovery),^18^^,^^19^ T2∗, T2-weighted imaging, early gadolinium enhancement, and late gadolinium enhancement (LGE) imaging. LGE images were obtained 10 to 15 minutes after administration of 0.13 mmol/kg of a gadolinium-based contrast agent (Gadoterate meglumine, Dotarem, Guerbet; or Gadodiamide, Omniscan, GE Healthcare).

### Image analysis

CMR images were analyzed using the CVI^42^ software (Circle Cardiovascular Imaging Inc) and MC-ROI, a dedicated in-house software (programmed in IDL, version 8.7, L3Harris Geospatial).^14^^,^^20^ IS was derived from LGE as the volume of myocardium with a signal intensity >5 SDs higher than the mean signal intensity in the remote region of interest (ROI). In patients with an old MI (defined as areas of LGE with no evidence of associated surrounding edema on T2-weighted imaging or T1 mapping), the mass of the old infarct was excluded. MVO was defined as a hypointense core inside an area of LGE and was manually delineated on short-axis slices. The presence of IMH was defined on T2-weighted imaging or on T2∗ maps.^14^^,^^16^ AAR was derived from T1-mapping^16^ and defined as the myocardium with T1 >2 SD above the mean T1 value of a remote myocardial ROI. Remote myocardial ROI was drawn 180^o^ away from the acutely infarcted segments when possible and with no LGE and normal wall motion. The area with LGE (including MVO) was always included in the AAR.

Good LV coverage with T1 maps was achieved for each patient (7 ± 2 slices per patient), resulting in 1,487 slices overall. Each slice was divided into 6 segments; of these, 10.3% (918 of 8,922) were excluded because of artifacts. Infarct, and remote and noninfarct T1 were derived from T1-maps based on the concept detailed in Central Illustration and Supplemental Figures 1 to 3. Infarcted and noninfarcted segments on T1-maps were defined by the presence or absence of LGE, respectively, on anatomically matching segments of delayed enhancement imaging. T1 >1,250 ms was considered to be abnormal and represents acute myocardial injury as previously published by our group,^14^ and is >2 SD above our normal range at 3.0-T (T1 = 1,184 ± 30 ms).

#### Angiographic analysis

TIMI (Thrombolysis In Myocardial Infarction) flow pre and post PPCI and myocardial blush grade were derived as described previously.^17^

#### MACE

Clinical outcomes data were prospectively collected from patients at their research visits, annual telephone calls, and from electronic patient records and general practitioners. MACE was defined as a composite endpoint consisting of cardiac death, new diagnosis of heart failure, survived cardiac arrest, and sustained ventricular arrhythmia requiring therapy. Heart failure was defined as the new occurrence of symptomatic fluid overload for which diuretic agents were administered in patients with left ventricular ejection fraction (LVEF) <50% and/or raised levels of natriuretic peptides.^17^ Any events before the acute CMR scan were excluded from analysis.

### Statistical analysis

Normality of data was determined using Kolmogorov-Smirnov test. Normally distributed data are presented as mean ± SD; nonparametric data as median (IQR). Statistical comparisons for paired and unpaired samples were performed using Student’s *t*-test, Wilcoxon signed rank test, or the Mann-Whitney *U* test, as appropriate. Categorical data and frequencies were compared with chi-square or Fisher’s test as appropriate. Segmental analysis was averaged on a per-subject basis before any interindividual and group comparisons to remove bias from clustering of segments within each subject. Correlation between variables was assessed using Pearson’s product-moment correlation coefficient (*r*) or Spearman’s rank correlation coefficient (ρ) as appropriate, depending on the normality assumption.

Survival analysis and endpoint comparison between groups were performed with the Cox regression analysis for the calculation of HR with 95% CI. Kaplan-Meier curves with log-rank test were constructed. Multicollinearity of variables included in the final model used was assessed using variance inflation factor analysis. The proportional-hazards assumption was met*.* Models of Cox regression multivariate analysis were created to explore the incremental predictive value of different CMR indices. CMR variables and clinical factors found to be significant univariate predictors of MACE with and without the novel T1 indices were included in the baseline model. The goodness of fit, predictive performance, discrimination, and risk classification of models with T1 indices were tested with: 1) concordance statistic (C-statistic); 2) Brier score; 3) Net-Reclassification Index (NRI); and 4) Integrated Discrimination Index (IDI) for censored data.

Statistical analyses were performed using SPSS (version 25, IBM Corp), Stata (version 16, StataCorp LLC), and R statistical software (version 4.0.5, RStudio, Inc, version 1.3); *SurvIDINRI* package was used on R Studio for the calculation of C-statistic, Brier score, NRI, and IDI. All statistical tests were 2-tailed, with values of *P* < 0.05 considered significant.

## Results

### Baseline clinical characteristics

A total of 221 patients with acute STEMI (age 61 ± 11 years; 87% male) were prospectively recruited; 5.4% of patients had a history of MI. Pain-to-balloon (ischemic) time was a median of 193 (IQR: 123-298) minutes. TIMI flow grade 3 was achieved after PPCI in 86% of patients.

Patients underwent an acute CMR scan at a median of 2 (IQR: 1-2) days after PPCI. Patients suffered significant myocardial injury, with a median peak troponin rise of 50 ng/dL (normal <0.04 ng/dL) and a mean LVEF of 47% ± 9% (Table 1). Most patients were treated with optimal medical treatment, with >98% of patients receiving dual antiplatelet therapy, statin, and either one of angiotensin-converting enzyme inhibitor or angiotensin receptor blocker; 93% of patients were discharged on a beta blocker (Supplemental Table 1). A total of 152 (69%) patients returned for a follow-up CMR scan and blood sampling at 6 months (Supplemental Methods).

**Table 1:** Baseline Demographics, Procedural, Angiographic and Investigations Data

	All Cohort (N = 219)	High Noninfarct T1 (n = 100)	Normal Noninfarct T1 (n = 119)	*P* Value
Age, y	61 ± 11	61 ± 11	61 ± 11	0.669
Male	86.9	82	90.8	0.072
BMI, kg/m^2^	27.6 ± 4.1	27.7 ± 4.2	27.5 ± 4	0.817
TIMI risk score	2 (1-4)	3 (2-6)	2 (1-6)	0.215
Smoking history	66.1	64	67.2	0.669
Hypertension	43.9	41	46.2	0.495
Diabetes	18.6	19	17.6	0.861
Hypercholesterolemia	38.0	40	36.1	0.578
Family history of CAD	41.6	36	46.2	0.133
Previous myocardial infarction	5.4	3.0	7.6	0.232
Previous PCI	4.5	2.0	6.7	0.115
Peripheral vascular disease	1.8	1	2.5	0.627
COPD or asthma	10.9	11	10.9	1.000
ACEI or ARB use before STEMI	21.7	24.0	20.2	0.516
Beta-blocker use before STEMI	9.5	8.0	10.1	0.644
Statin use before STEMI	20.8	20.0	21.0	0.869
Systolic BP on admission, mm Hg	131 ± 26	126 ± 25	136 ± 26	**0.010**
Heart rate on admission, beats/min	79 ± 19	82 ± 19	76 ± 18	**0.023**
Ischemic time, min	193 (123-298)	174 (110-293)	196 (138-298)	0.175
Late presenter, 6-12 h	19.5	19.2	19.3	1.000
Aspirin pre PPCI	99.1	98.0	99.9	0.207
LAD culprit	46.6	60	35.3	**<0.001**
MVD, >1 vessel disease	30.3	23	37	**0.028**
TIMI flow pre PPCI = 0	70.1	73	68.1	0.460
Thrombus score >2	83.3	85.6	79.9	0.451
TIMI flow post PPCI = 3	86.0	85	87.4	0.694
Myocardial blush grade <2	17.2	17.6	17.1	1.000
Troponin peak level, ng/L	50 (21-131)	59 (35-216)	45 (16-106)	**0.012**
Creatinine level on admission, μmol/L	78 ± 19	75 ± 18	81 ± 19	**0.033**
Peak monocyte count, × 10^9^/L	0.91 (0.68-1.20)	0.94 (0.73-1.21)	0.87 (0.65-1.20)	0.188
Peak neutrophil count, × 10^9^/L	8.4 ± 2.6	8.6 ± 2.5	8.2 ± 2.6	0.303
Peak C-reactive protein, mg/L	5.9 (2.4-15.8)	7.2 (2.8-19.1)	5.2 (1.9-13.2)	0.187
ST-segment resolution >70% on ECG	75.7	74	76.8	0.747

Values are mean ± SD, %, or median (IQR), unless otherwise indicated.

ACEI = angiotensin converting enzyme inhibitor; ARB = angiotensin receptor blocker; BMI = body mass index; BP = blood pressure; CAD = coronary artery disease; COPD = chronic obstructive pulmonary disease; ECG = electrocardiogram; LAD = left anterior descending artery; MVD = multivessel disease; PCI = percutaneous coronary intervention; PPCI = primary percutaneous coronary intervention; STEMI = ST-segment elevation myocardial infarction; TIMI = Thrombolysis In Myocardial Infarction.

### Acute tissue characteristics of the infarcted myocardium

The median IS was 22% (IQR: 13%-33%). MVO and/or IMH were present in 53% of cases. Infarct T1 was available in 90% of the cohort and the mean was significantly elevated at 1,366 ± 68 ms. Infarct T1 was lower in patients with large MVO (>1.55% of myocardium): (1,355 ± 63 ms vs 1,376 ± 70 ms; *P =* 0.032). The area of MVO had a significantly lower T1 than the surrounding infarcted area (1,293 ± 74 ms vs 1,369 ± 63 ms; *P* < 0.001).

### Acute tissue characteristics of the noninfarcted myocardium

Overall, 46% of patients had significantly elevated mean T1 >1,250 ms in the entire noninfarcted myocardium. When compared with the patients with normal mean T1 in the noninfarcted myocardium, those with high noninfarct T1 had significantly lower LVEF (50% ± 7% vs 44% ± 9%; *P* < 0.001), larger IS (median: 20% vs 28%; *P* < 0.001), larger AAR (36% ± 12% vs 47% ± 13%; *P* < 0.001), and larger MVO volume (median: 0% vs 1.2%; *P* < 0.007), as well as a numerically higher prevalence of IMH (47% vs 60%; *P =* 0.109) (Table 2). In terms of clinical characteristics, patients with high noninfarct T1 had a significantly higher proportion of left anterior descending coronary artery infarctions (35% vs 60%; *P* < 0.001) but lower prevalence of multivessel disease (37% vs 23%; *P =* 0.028).

**Table 2:** Acute CMR Findings Stratified According to Higher and Lower T1 in Infarcted and Noninfarcted Myocardium

Acute CMR Findings (Median 2 d)	Patients (N = 219)	Infarct T1			Noninfarct T1		
		Higher T (≥1,300 ms) (87% of Cohort)	Lower T1 (<1,300 ms) (13% of Cohort)	*P* Value	High T1 (>1,250 ms) (46% of Cohort)	Normal T1 (≤1,250 ms) (54% of Cohort)	*P* Value
Acute LVEDVI, mL/m^2^	80 ± 17	81 ± 15	82 ± 19	NS	83 ± 17	78 ± 16	**0.016**
Acute LVESVI, mL/m^2^	43 ± 13	44 ± 12	44 ± 16	NS	47 ± 14	39 ± 11	**<0.0001**
Acute LVEF, %	47 ± 9	47 ± 9	47 ± 9	NS	44 ± 9	50 ± 7	**<0.00001**
Acute RVEDVI, mL/m^2^	65 ± 14	65 ± 14	67 ± 17	NS	63 ± 13	67 ± 15	NS
Acute RVESVI, mL/m^2^	29 ± 10	29 ± 10	30 ± 12	NS	28 ± 9	30 ± 11	0.082
Acute RVEF, %	56 ± 8	56 ± 8	56 ± 9	NS	57 ± 9	56 ± 8	NS
Acute AAR, % of LV mass	41 ± 14	44 ± 13	35 ± 14	**0.005**	47 ± 13	36 ± 12	**<0.00001**
Acute IS, % of LV mass	22 (13-33)	24 (16-34)	22 (13-29)	NS	28 (16-38)	20 (11-27)	**<0.00001**
MVO, % of LV mass	0.57 (0.00-1.19)	0.86 (0.00-3.38)	1.23 (0.00-6.31)	NS	1.16 (0-5.22)	0 (0-2.17)	**0.007**
MVO presence, % of patients	52.8	57.9	59.3	NS	60.2	47.4	0.080
IMH presence, % of patients	52.3	58.8	56.0	NS	59.7	47.1	NS
RV infarct, % of patients	25.5	26.9	25.9	NS	26.2	24.7	NS
Global T1, ms	1,273 ± 50	1,287 ± 43	1,227 ± 29	**<0.00001**	1,311 ± 38	1242 ± 36	**<0.00001**
Infarct T1, ms	1,366 ± 68	1,384 ± 55	1,262 ± 36	**<0.0001**	1,387 ± 62	1349 ± 69	**<0.00001**
Infarct T1 adjusted for large MVO, ms	1,372 ± 67	1,389 ± 56	1,271 ± 33	**<0.00001**	1,394 ± 59	1,352 ± 68	**<0.00001**
Noninfarct T1, ms	1,245 ± 43	1,253 ± 39	1,217 ± 38	**<0.00001**	1,283 ± 28	1,214 ± 25	**<0.00001**
Remote T1, ms	1,203 ± 46	1,209 ± 45	1,182 ± 43	**0.003**	1,232 ± 41	1,179 ± 34	**<0.00001**

Values are mean ± SD, median (IQR), or %. Values in **bold** indicate a *P* value <0.05.

AAR = area at risk; CMR = cardiac magnetic resonance; IMH = intramyocardial hemorrhage; IS = infarct size; LV = left ventricle; LVEDVI = indexed left ventricular end-diastolic volume; LVEF = left ventricular ejection fraction; LVESVI = indexed left ventricular end-systolic volume; MVO = microvascular obstruction; NS = nonsignificant *P* value; RV = right ventricle; RVEDVI = indexed right ventricular end-diastolic volume; RVEF = right ventricular ejection fraction; RVESVI = indexed right ventricular end-systolic volume*.*

### Acute tissue characteristics of the remote zone within the noninfarcted myocardium

The mean T1 value in the remote zone within the noninfarcted myocardium was 1,203 ± 46 ms (Table 2), which is significantly higher than the normal T1 values (1,184 ± 30 ms; *P* < 0.001). Twelve percent of patients had high remote T1 (>1,250 ms).

#### LV characteristics at 6-month follow-up

Mean LVEF improved significantly from baseline (47% to 53%; *P* < 0.001) and the final IS was significantly smaller (22% to 16%; *P* < 0.001) at 6 months post-STEMI. The myocardial salvage index was 61% ± 21% (Table 3).

**Table 3:** CMR Findings and NT-proBNP Levels at 6 Months Post-STEMI Stratified According to Higher and Lower T1 in the Noninfarcted Myocardium on the Acute Scan

CMR Findings at 6 Months	Patients With Follow-Up CMR (n = 152)	Acute Noninfarct T1		
		High (>1,250 ms) (47% of Cohort)	Normal (≤1,250 ms) (53% of cohort)	*P* Value
LVEDVI, mL/m^2^	84 ± 19^a^	86 ± 20	81 ± 18	0.115
LVESVI, mL/m^2^	40 ± 15^a^	44 ± 16	38 ± 14	**0.015**
LVEF, %	53 ± 9^a^	51 ± 8	55 ± 9	**0.002**
RVEDVI, mL/m^2^	71 ± 14^a^	70 ± 14	71 ± 14	0.541
RVESVI, mL/m^2^	29 ± 9^a^	29 ± 8	29 ± 9	0.649
RVEF, %	60 ± 7^a^	60 ± 7	60 ± 6	0.899
Final infarct size, %	16 (8-24)^a^	19 (10-28)	13 (8-25)	**0.005**
Myocardial salvage index, %	61 (45-76)	58 (42-72)	64 (46-78)	0.258
Change in LVEDV vs baseline, %	1.6 (−8.1 to 11.2)	2.8 (−8.3 to 14)	0.9 (−5.4 to 4.4)	0.267
Significant adverse remodeling, frequency in %	14	9	15	0.226
Absolute change in LVEF	5 (0-10)	7 (2-12)	3 (−1 to 8)	**0.011**
Blood test findings at 6 mo				
NT-proBNP, pg/mL	198 (88-398)	290 (103-523)	170 (61-312)	**0.008**

Values are mean ± SD, median (IQR), or %.

NT-proBNP = N-terminal pro–B-type natriuretic peptide; other abbreviations as in Tables 1 and 2.

a *P* < 0.001 when compared with acute CMR scan findings in Table 2. Significant adverse remodeling is defined as ≥20% increase in LVEDV. Values in bold indicate a value of *P* < 0.05.

### Predictive power of acute T1 indices for short-term (6-month) outcomes

**Figure 2:**
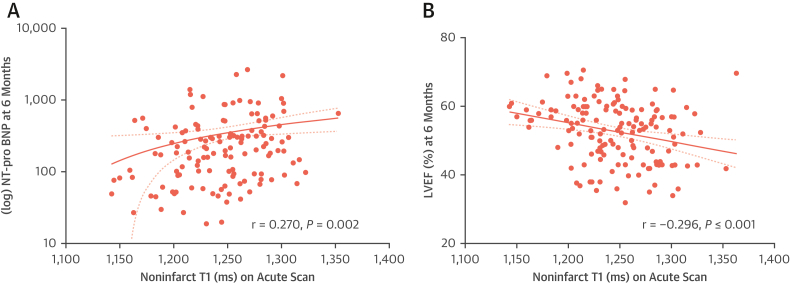
Correlation of Noninfarct T1 on Acute CMR Scan With LV Systolic Function and Natriuretic Peptide Levels at 6 Months Correlation between noninfarct T1 on acute scan with **(A)** N-terminal pro–B-type natriuretic peptide (NT-proBNP) (pg/L) and **(B)** left ventricular ejection fraction (LVEF) (%) at 6 months. LV = left ventricular; other abbreviation as in Figure 1

Acute infarct T1 was not significantly correlated with LVEF or N-terminal pro–B-type natriuretic peptide (NT-proBNP) at 6 months. In contrast, acute noninfarct T1 and remote T1 significantly correlated with LVEF (*r* = −0.30 and *r* = −0.35, respectively; both *P <* 0.001) and NT-proBNP levels (*r* = 0.27; *P =* 0.002 and *r* = 0.24; *P =* 0.007, respectively) at 6 months (Figure 2). When compared with patients with normal noninfarct T1, those with high noninfarct T1 on the acute scan had lower LVEF (55% ± 9% vs 51% ± 8%; *P =* 0.005), larger final IS (13% vs 19%; *P =* 0.005), and higher NT-proBNP levels (median: 170 vs 290 pg/mL; *P =* 0.008) at 6 months (Table 3). Similarly, patients with high remote T1 also had lower LVEF (54% ± 9% vs 47% ± 8%; *P =* 0.001), larger final IS (13% vs 27%; *P* < 0.001), and higher NT-proBNP levels (median: 186 vs 319 pg/mL; *P =* 0.022) at 6 months.

### Long-term MACE

Two patients were lost to follow-up. Median follow-up duration was 4 (IQR: 2.5-6.0) years. Outcome analysis was censored to 2,700 days, as very few patients had a greater follow-up duration. During the follow-up period, 22 patients (10%) experienced a MACE; new diagnosis of heart failure occurred in 20 and ventricular arrhythmia and cardiac death in 1 each.

### Univariate Cox regression analysis of long-term MACE

#### Clinical and procedural factors

Previous MI, longer ischemic time, and higher peak troponin were significantly associated with MACE (all *P <* 0.01), whereas peak monocyte count trended toward significance *(P =* 0.087) (Table 4).

**Table 4:** Univariate Cox Regression Analysis

	HR (95% CI)	*P* Value
Clinical factors and angiographic indices at admission		
Age	1.029 (0.989-1.070)	0.158
Smoking history	0.595 (0.257-1.070)	0.226
Hypertension	1.400 (0.606-3.237)	0.431
Diabetes	0.897 (0.303-2.652)	0.844
Previous history of MI	5.653 (1.867-17.114)	**0.002**
Ischemic time per minute	1.001 (1.000-1.003)	**0.008**
LAD culprit	1.068 (0.461-2.474)	0.878
Multivessel disease (>1 vessel)	0.706 (0.26-1.914)	0.494
Thrombus score >2	1.107 (0.326-3.763)	0.871
TIMI flow post PPCI <3	1.513 (0.555-4.122)	0.418
Myocardial blush grade post PPCI <2	1.199 (0.403-3.565)	0.744
Peak troponin rise, per unit increase	1.002 (1.001-1.003)	**0.005**
Peak monocyte count, per unit increase	1.993 (0.904-4.391)	**0.087**
>70% ST-segment resolution at discharge	0.516 (0.216-1.230)	0.136
Prescription of GDMT at discharge	0.536 (0.158-1.182)	0.353
Prescription of GDMT at 6 mo	3.359 (0.448-25.17)	0.238
Prescription of GDMT at final follow-up	0.889 (0.375-2.112)	0.791
Acute CMR markers		
LVEF per 1% increase	0.951 (0.905-1.00)	**0.048**
LVEF ≤40%	3.554 (1.506-8.385)	**0.004**
RVEF per 1% increase	1.028 (0.971-1.088)	0.339
Area at risk per 1% increase	1.035 (0.996-1.077)	**0.083**
Infarct size per 1% increase	1.028 (0.997-1.060)	**0.082**
Infarct size >17%	3.529 (1.033-12.055)	**0.044**
RV infarct presence	0.876 (0.289-5.623)	0.114
MVO presence	3.734 (1.248-11.172)	**0.018**
IMH presence	2.161 (0.830-5.623)	0.114
Acute T1 indices		
Global T1	1.002 (0.994-1.011)	0.580
Infarct T1 per ms	0.993 (0.986-0.999)	**0.035**
Infarct T1 <1,300 ms	3.114 (1.192-8.133)	**0.020**
Noninfarct T1 per ms	1.009 (1.000-1.018)	**0.056**
Noninfarct T1 per 10 ms	1.094 (1.000-1.195)	**0.056**
Noninfarct T1 >1,250 ms (>2 SD)	2.534 (1.033 -6.219)	**0.042**
Remote T1 per ms	1.008 (0.999-1.017)	**0.083**
Remote T1 >1,250 ms (>2 SD)	2.223 (0.818-6.037)	0.112

Univariate Cox regression analysis of clinical, routine CMR indices and T1 biomarkers. Values in **bold** are *P* < 0.10 and subsequently included in the multivariate Cox regression in Table 5.

GDMT = Guideline directed medical therapy (beta-blocker and angiotensin-converting enzyme inhibitor or angiotensin receptor blocker); MI = myocardial infarction; other abbreviations as in Tables 1 and 2.

#### Conventional CMR markers

LVEF (HR: 0.951 per 1% increase in LVEF; *P =* 0.048), IS (HR: 1.028 per 1% increase in IS; *P =* 0.080), and MVO (HR: 1.109 per 1% increase in MVO: *P =* 0.005) were associated with MACE. When dichotomized, LVEF <40% (HR: 3.554; *P =* 0.004), IS >17% (HR: 3.529; *P =* 0.044), and the presence of MVO (HR: 3.714; *P =* 0.018) were associated with MACE (Table 4).

#### T1 indices

Infarct T1 was significantly inversely associated with MACE (HR: 0.993 per 1-ms increase; *P =* 0.035). Most (87%) patients had a significantly high infarct T1 (≥1,300 ms), whereas 13% of patients had an average infarct T1 <1,300 ms, referred to as “lower infarct T1” from here on. Lower infarct T1 was associated with a 3-fold increased risk of MACE (HR: 3.114; 95% CI: 1.192-8.133; *P =* 0.020). Noninfarct T1 (HR: 1.009 per 1-ms increase; *P =* 0.056) and remote T1 (HR: 1.008 per 1-ms increase; *P =* 0.083) were associated with MACE. A high noninfarct T1 was associated with a 2.5-fold increased risk of MACE (HR: 2.534; 95% CI: 1.033-6.219; *P =* 0.042).

### Multivariate Cox regression analysis of MACE

#### CMR model

Both acute infarct T1 and noninfarct T1 showed incremental value beyond conventional CMR markers (LVEF, IS, MVO) in predicting long-term MACE. When these T1 indices were added to a model of multivariate Cox regression (both nonstepwise and stepwise), only MVO (*P =* 0.038), infarct T1 (*P =* 0.001), and noninfarct T1 (*P =* 0.001) remained as independent predictors of MACE (Table 5, Supplemental Table 2). When dichotomized, lower infarct T1 (*P =* 0.001) and high noninfarct T1 (*P =* 0.027) were the only independent predictors of MACE). Remote myocardial T1 also demonstrated a similar but weaker ability to predict MACE in such models (as continuous variable: *P =* 0.019; but when dichotomized: *P =* 0.234) (Table 5, Supplemental Table 2).

**Table 5:** Multivariate Cox Regression Analysis of Conventional CMR and Novel T1 Indices

	HR (95% CI)	*P* Value
Model 1		
Acute LVEF, per 1% increase	1.017 (0.954-1.084)	0.608
MVO presence	2.829 (0.898-8.909)	0.076
Noninfarct T1, per 1 ms	1.024 (1.010-1.039)	**0.001**
Infarct T1, per 1 ms	0.987 (0.979-0.994)	**0.001**
Model 2		
Acute LVEF **≤**40%	1.482 (0.538-4.083)	0.447
MVO presence	2.470 (0.799-7.630)	0.116
Noninfarct T1, per 1 ms	1.020 (1.006-1.034)	**0.005**
Infarct T1, per 1 ms	0.988 (0.981-0.995)	**0.001**
Model 3		
Acute LVEF ≤40%	2.347 (0.899-6.127)	0.081
MVO presence	2.013 (0.646-6.273)	0.228
Noninfarct T1 >1,250 ms	3.643 (1.169 -11.357)	**0.026**
Infarct T1 <1,300 ms	5.982 (2.077-17.277)	**0.001**

Multivariate Cox regression analysis to establish CMR variables predictive of clinical outcomes. Models 1-2 contain T1 as continuous variable. Model 3 uses T1 cutoff as a categorical variable. Values in **bold** are *P* < 0.05 and were considered to be independent predictors of clinical outcomes. See Supplemental Materials for further multivariate Cox regression models.

Abbreviations as in Table 2.

#### Holistic model incorporating clinical and CMR characteristics

Both infarct T1 and noninfarct T1 remained as significant independent predictors of MACE in multivariate Cox regression models consisting of clinical variables (age, previous MI, ischemic time, and peak troponin) and conventional CMR indices (Supplemental Tables 3 and 4).

### Discrimination and risk reclassification for MACE using novel T1 indices

Beyond the conventional CMR model, the addition of infarct T1 and noninfarct T1 significantly improved the prediction and reclassification of MACE. When added to a CMR model consisting of LVEF, IS, and MVO, the C-statistic improved from 0.67 ± 0.07 to 0.76 ± 0.06 and the integrated Brier score improved from 0.076 to 0.064. In a reclassification analysis, the inclusion of noninfarct T1 and infarct T1 resulted in an NRI of 40% (95% CI: 12%-64%; *P =* 0.007) and an IDI of 16% (95% CI: 7%-31%; *P <* 0.001), all indicating improved predictive performance of the T1 indices (Table 6). Similarly, noninfarct T1 and infarct T1 significantly improved the reclassification performance of a holistic model consisting of clinically important factors and conventional CMR markers (Supplemental Table 5).

**Table 6:** Comparisons of Models of Conventional CMR Indices With and Without Novel T1 Indices in Predicting Long-Term MACE After STEMI

Model	Predictors	C-statistic	Integrated Brier Score	Comparison of Models		Net Reclassification Index		Integrated Discrimination Improvement Index	
				Chi-Square Difference	*P* Value	Index	*P* Value	Index	*P* Value
A	Acute LVEF MVO presence Infarct size	0.67 ± 0.07	0.076	N/A					
B	Acute LVEF MVO presence Infarct size Infarct T1 Noninfarct T1	0.76 ± 0.06	0.064	Model A vs B					
				15.52	<0.001	39.8% (95% CI: 12.2%-64.4%)	0.007	16.4% (95% CI: 6.8%-30.9%)	<0.001
C	Acute LVEF <40% MVO presence Infarct size	0.69 ± 0.06	0.075	N/A					
D	Acute LVEF <40% MVO presence Infarct size Infarct T1 Noninfarct T1	0.77 ± 0.06	0.064	Model A vs D					
				17.32	<0.001	50.4% (CI 17.7%-68.3%)	0.013	18.7% (CI 6.5%-32.4%)	<0.001
				Model C vs D					
				13.21	0.001	42.3% (CI 1.4%-66.2%)	0.027	16.1% (CI 4.7%-30.1%)	<0.001

Comparison of the ability of models with conventional CMR indices (A, C) and models with T1 indices added (B, D) to predict clinical outcomes.

C-statistic = concordance statistic; MACE = major adverse cardiac events; N/A = not applicable; other abbreviations as in Tables 1 and 2.

## Discussion

Our study is the first to assess the long-term prognostic value of the acute changes in both the noninfarcted and infarcted myocardium immediately after STEMI. Our novel contributions are as follows:

1. A significant acute response in the noninfarcted myocardium, defined as high T1 values on CMR, was associated with significantly lower LVEF and higher levels of natriuretic peptides at 6 months and long-term MACE.
2. Noninfarct and infarct T1 are significant independent predictors of long-term MACE and offer better reclassification and significantly improved predictive performance beyond conventional CMR markers (LVEF, IS, and MVO).

**Figure 3:**
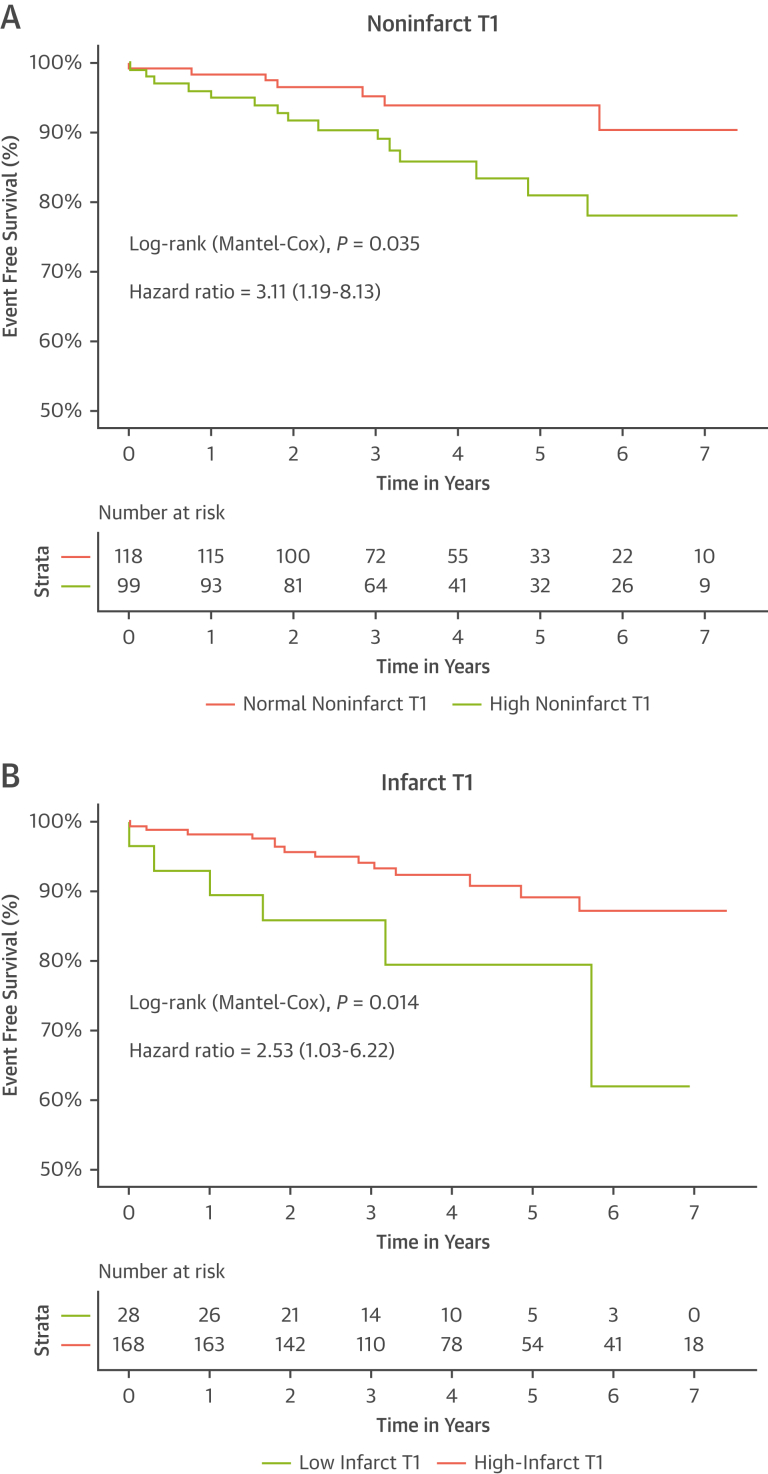
Noninfarct T1 and Infarct T1 on Acute CMR Scan Predict Long-Term MACE Kaplan-Meier survival curves showing the differences in survival from MACE between **(A)** patients with high T1 (>1,250 ms) and normal T1 (<1,250 ms) in the noninfarcted myocardium, **(B)** patients with lower (<1,300 ms) and high (≥1,300 ms) T1 values in the infarcted myocardium. Abbreviations as in Figure 1.

Infarcted myocardial tissue undergoes a high degree of inflammatory changes and exhibits significantly increased T1 times, secondary to the expansion of both intracellular and extracellular volume from myocardial necrosis, edema, and the ensuing inflammation.^11^^,^^12^^,^^14^^,^^21^ The core of the infarcted segment often has a lower T1 value caused by the presence of MVO and/or IMH, and has been shown to be associated with poor outcomes at a median of 2.3 years post-STEMI.^15^ We extended this finding by showing that a lower T1 in the entire infarcted segment is predictive of long-term MACE at a median of 4 years. For every 10-ms decrease in infarcted myocardial T1, there is a 7% increased risk of adverse outcomes, whereas an overall lower infarct T1 (<1,300 ms) was associated with a 3-fold increase in risk of MACE (Table 4, Figure 3). Infarct T1 indices offered significant improvement in risk prediction beyond conventional markers, such as IS and presence of MVO (Table 5, Supplemental Table 2).

More interestingly, beyond the infarcted territory, remote myocardial injury and dysfunction post-MI have been described in animal and human studies.^3^, ^4^, ^5^, ^6^, ^7^, ^8^ The proposed mechanisms include activation of the innate immune system and inflammation.^4^ Remote myocardial inflammation is believed to be mediated mainly by a 5- to 6-fold increase in leukocytes, and is coupled with an upregulation of inflammatory mediators including cytokines, adhesion molecules, and matrix metalloproteinases.^3^^,^^4^ These may result in adverse LV remodeling, fibrosis, and systolic dysfunction.^8^

Increased remote myocardial T1 post-STEMI is known to be associated with adverse short-term outcomes, which we have also confirmed in this study, but its long-term prognostic implication had remained unclear.^6^^,^^7^ We have now shown that although the remote myocardial T1 is weakly associated with long-term MACE, a more complete assessment of the entire noninfarcted myocardium with T1-mapping provides a more powerful and independent prediction of long-term MACE (Tables 4 and 5, Supplemental Table 2). In particular, for every 10-ms increase in noninfarct myocardial T1, the risk of long-term MACE increased by 9%. This suggests that the assessment of just a small remote zone of the noninfarcted myocardium is less comprehensive. Equally, neither the acute IS nor AAR (which relies on the assumption that remote myocardium is normal) were independent predictors of MACE in multivariate Cox regression models containing the noninfarct and infarct T1.

An acutely high remote T1 may be a surrogate marker for a large insult to the LV myocardium in STEMI. In our study, the abnormal response in the noninfarcted myocardium was associated more frequently with left anterior descending coronary artery infarctions, larger acute IS, and higher peak troponin level (Tables 1 and 2). Multivessel disease was not a predictor of clinical outcomes and, more interestingly, was more prevalent among those with normal noninfarct T1 than in those with high noninfarct T1 (37% vs 23%; *P =* 0.028). Thus, it is unlikely that resting ischemia from nonculprit vessels contributed significantly to the severe elevation observed in the noninfarcted myocardium.

Although the fraction of global myocardium with high T1 is positively correlated with inflammatory biomarkers, only remote T1 showed weak relationship with neutrophil counts and trend correlations with monocytes and C-reactive protein level post PPCI (Supplemental Table 6). The lack of correlations within either the infarct and noninfarct tissue T1 is likely caused by the heterogeneity of these tissue classes characterized by various competing mechanisms affecting the measured T1 (eg, MVO core in the infarcted area). In contrast, the remote myocardium T1 changes were found to be a better overall indicator of the systemic inflammatory burden. This is in keeping with preclinical and clinical observations that remote myocardial injury is related to the ischemic insult and the ensuing immune response and inflammation.^3^^,^^4^^,^^7^^,^^22^

The novelty of establishing the prognostic power of noninfarcted myocardial injury is best seen in the multivariate Cox regression analysis. In all the models consisting of conventional CMR indices (LVEF, IS, MVO), noninfarct myocardial T1 was an independent predictor of outcomes (Table 5, Supplemental Table 2). Indeed, when the models include both infarct and noninfarct myocardial T1, the performance of CMR in predicting adverse outcomes improved significantly (Table 6). Furthermore, the T1 indices significantly improved the predictive ability of a model containing conventional CMR indices and traditional clinical factors (Supplemental Tables 3 and 4). These findings challenge the current consensus that IS and LVEF should be the main prognostic indices in patients after STEMI,^2^ and make a case to also include the assessment of the pan-myocardial response using T1 mapping. For instance, in our study 9 of 45 (20%) patients with LVEF <40% and 12 of 174 (7%) patients with LVEF ≥40% developed a long-term MACE. However, high noninfarct T1 was present in 7 of 12 (58%) of the patients with LVEF >40% who experienced an MACE; thus, if confirmed in larger trials, novel T1 indices may be used to reclassify patients otherwise labeled as low risk, to aggressively treat and maintain optimal medical therapy to reduce the risk of long-term MACE.

Our new findings build on previous reports of an association between remote myocardial changes after STEMI and adverse LV remodeling and the expansion of extracellular matrix at short-term follow-up,^6^^,^^7^^,^^9^ and demonstrate a strong association with the development of heart failure at long-term. Interestingly in our study, although the significantly abnormal T1 values in either infarcted or noninfarcted territories immediately after STEMI predicted the development of heart failure at long-term, they were not associated with adverse LV remodeling at 6 months (Table 3). Further work is required to test the performance of native T1 indices against other acute CMR indices in predicting outcomes post-STEMI, including remote extracellular volume^9^ and remote myocardial strain,^23^ which currently remain exploratory endpoints.^2^ Similarly, the influence of infarcted myocardium on the functional recovery of the peri-infarcted area (noninfarcted myocardium) may provide further insights.^24^

We propose that T1-mapping could be included for a more holistic and directly quantitative assessment of the global myocardial response and risk prediction post-STEMI. It would therefore be of clinical interest to test the prognostic benefit of identifying such at-risk patients early after STEMI and offering them aggressive optimization of existing medical therapy as well as trials of immunomodulation.

### Study limitations

This is a single-center study of patients with a lower-risk clinical profile on admission (no cardiogenic shock) and only those who were stable for a CMR scan on day 2, and therefore carries a potential selection bias. However, we believe that the discovery of novel T1 indices to further stratify the long-term risk of such patients is of significance. Scanning at day 2 may miss detecting the bimodal intensity in edema associated with post-reperfusion (first few hours) and inflammatory response (days 4-7). However, CMR scanning of patients post STEMI at approximately 2 days was an accepted practice at the time of our study protocol design and remains the most practical and clinically relevant time point for patients in the United Kingdom, as it coincides with their hospital discharge.^15^^,^^25^ Capturing T1 signals at either peak of the bimodal edema may provide stronger associations with outcomes, but awaits further research. Although T1 maps were acquired at 3.0-T, the use of thresholding to establish an abnormal T1 (>2 SD) can be performed in 1.5-T scanners because Shortened Modified Look-Locker Inversion recovery sequences have been validated in both settings.^18^ A higher number of events would be needed to firmly conclude associations with CMR imaging markers; however, the strength of our study lies in its prospective design with serial and timely long-term follow-up of each patient (median: 4 years). Moreover, we studied clinical outcomes of significance (heart failure, ventricular arrhythmias or cardiac arrest, cardiac death), and the rate of events is in line with real-world data, revealing strong statistical associations with CMR markers in univariate and multivariate Cox regression models. High multicollinearity was excluded by our analysis (Supplemental Table 7). However, some degree of collinearity between the CMR variables in these models may reduce the accuracy of our findings and thus reproducibility studies with larger sample sizes and longer follow-up are needed. It is impossible to exactly match LGE and T1 images; however, to minimize the differences, T1-maps and LGE images were obtained at the same slice position in the scanner and analyzed by experienced operators. Noninfarcted myocardium, defined as the area without LGE, includes both the salvageable myocardium as well as the myocardium supplied by nonculprit arteries. We did not differentiate the T1 changes in these 2 compartments for 2 reasons. First, it is impossible to accurately delineate the true myocardial area subtended by a coronary artery. Second, because we observed that the remote T1 is elevated and is associated with outcomes, the derivation of AAR assuming a normal remote region is prone to errors. The exact pathophysiology behind the alterations in infarct and noninfarct T1 in these patients cannot be ascertained without direct histopathology correlates; however, it is widely considered to represent acute myocardial edema in this setting, likely associated with myocardial necrosis and the resulting inflammation.^11^^,^^12^^,^^26^ Although the calculation of noninfarct T1 requires the manual matching of T1-maps and LGE images, it has the potential to be made fully automated with the use of artificial intelligence in CMR.^27^ Our study did not use T2-mapping, which has been shown to detect subtle edema in the remote myocardium post-STEMI,^28^ but there is strong evidence that T1-mapping is a suitable alternative to detect myocardial edema.^11^^,^^13^

## Conclusions

The global acute responses post-STEMI in both infarcted and noninfarcted myocardium quantified on CMR T1-mapping are independent predictors of long-term MACE, beyond conventional markers such as LVEF, IS, and MVO. These insights may provide new opportunities for treatment and risk stratification of patients with acute STEMI.

Perspectives **COMPETENCY IN MEDICAL KNOWLEDGE:** Our findings have implications for future research and clinical practice. We show that acute STEMI produces a global myocardial response in both infarcted and noninfarcted regions, as detected on CMR T1-mapping. These T1 indices are associated with long-term adverse cardiac outcomes, mainly that of new-onset heart failure, and are more predictive beyond conventional indices such as LVEF, IS, and MVO. Therefore, it holds much promise in our future attempts to better detect the pan-myocardial injury in STEMI, monitor response to treatment, and offer prognostication.

**TRANSLATIONAL OUTLOOK:** This study shows that the assessment of injury in both infarcted and noninfarcted myocardium immediately after reperfused STEMI using noncontrast T1-mapping on CMR offers independent and incremental prediction of long-term adverse clinical outcomes, mainly that of new-onset heart failure. It would be desirable to design trials in which patients with significant noninfarct myocardial injury after STEMI are started on optimal medical therapy irrespective of the LVEF to see if it would reduce the risk of heart failure in the long-term.

## Funding Support and Author Disclosures

The OxAMI study is supported by the British Heart Foundation (BHF) Centre of Research Excellence (CRE) Oxford (RE/13/1/30181), and the National Institute for Health Research (NIHR) Oxford Biomedical Research Centre. Dr Shanmuganathan is supported by the Alison Brading Memorial Graduate Scholarship in Medical Science, Lady Margaret Hall, University of Oxford. Dr Burrage was supported by a BHF Clinical Research Training Fellowship (FS/19/65/34692). Prof Channon is funded by a BHF Chair award (CH/16/1/32013). Prof Ferreira has received support from the BHF, BHF CRE Oxford, and NIHR Oxford BRC. Prof Piechnik and Dr Zhang have received support from the BHF CRE Oxford (RE/18/3/34214). Prof Piechnik has patent authorship rights for U.S. patent 9285446 B2 (systems and methods for Shortened Look-Locker Inversion Recovery [Sh-MOLLI] cardiac gated mapping of T1), granted March 15, 2016; all rights transferred to Siemens Medical. The funders were not involved in the design and conduct of the study, in the collection, analysis, and interpretation of the data, and in the preparation, review, or approval of the paper*.* All other authors have reported that they have no relationships relevant to the contents of this paper to disclose.
