# Supplementary material for: Acute Response in the Noninfarcted Myocardium Predicts Long-Term Major Adverse Cardiac Events After STEMI
Source: JACC Cardiovasc Imaging. 2023 Jan;16(1):46–59. doi: 10.1016/j.jcmg.2022.09.015 (PMC9834063; doi:10.1016/j.jcmg.2022.09.015)
Supplement: Supplemental Data [file mmc1.docx]

**SUPPLEMENTARY DATA**

**Supplementary Table 1. Cardiac medications on hospital discharge and at 6 months.**

| **Medications** | **Frequency on discharge (%)** | **Frequency at 6 months (%)** |
| --- | --- | --- |
| Aspirin | 98 | 95 |
| Clopidogrel or Ticagrelor | 99 | 96 |
| Beta blocker | 93 | 88 |
| ACE-i or ARB | 99 | 95 |
| Statin | 99 | 96 |

ACE-i = Angiotensin converting enzyme inhibitor, ARB = Angiotensin receptor blocker

**SUPPLEMENTARY METHODS**

**ECG and blood tests:** Serial blood samples and ECGs were obtained on patient admission to hospital and at 6 months. White blood cell differential count, Troponin and C-reactive protein levels were serially measured, at clinically relevant times, during the index admission. Peak values are presented in Table 2. NT-pro BNP level was measured at 6 months.

**ShMOLLI T1 mapping image analysis on MC-ROI**: Endocardial and epicardial contours were placed using dedicated automated software (1) and manually checked for errors by two experts (AB and AM with over 5 and 3 years’ of experience, respectively) blinded to clinical data. Data were processed in single batch and checked for consistency (SKP) subject to further correction of contours (AM, AB). Apical slices affected by partial volume effects and slices where the outflow tract was visible were excluded from the study in all sequences. Short-axis images were divided into 6 equiangular segments with the anterior right ventricle–left ventricle junction as the reference point according to the American Heart Association (AHA) 16-segment model (see Supplementary Figure 1-3 below). Each colour map was assessed for the presence of artefacts using previously described core-lab protocols (2) . Segments affected by artefacts (breathing, ECG triggering, or other artefacts) were marked and excluded from the final analysis. Segments on T1 maps corresponding to LGE (infarcted myocardium) and no LGE (non-infarcted myocardium) were identified.

A region of interest (ROI) was accurately placed in the remote myocardium, defined as myocardium 180° degrees from the affected zone with no LGE, wall motion abnormalities or edema.

In slices with no LGE in the same patient, remote myocardial ROI was drawn in a similar position to the slices with LGE. In patients with large MVO (>1.55% of the myocardium) on LGE imaging, corresponding regions on T1 maps were demarcated. See Figure 1 for an illustration of this concept. Topological analysis of T1 maps was performed with threshold T1>1250ms considered to reflect acute myocardial injury, in line with the normal range for ShMOLLI in our 3T scanners (1124-1244ms) and previous work from our group (3).

As per local quality control protocol (artefact scoring system) segments on ShMOLLI T1-maps corresponding to LGE imaging with delayed enhancement on LGE imaging were scored as ‘0’; i.e. infarct. Segments without delayed enhancement on LGE imaging were scored as ‘1’, i.e. non-infarct. Segments with artefacts were scored as one of ‘2 = poor planning, 3 = motion (breathing or cardiac), 4 = missing ECG trigger, 5 = image acquisition and reconstruction artefacts, or 6 = unidentified artefact’.

**SUPPLEMENTARY FIGURES**


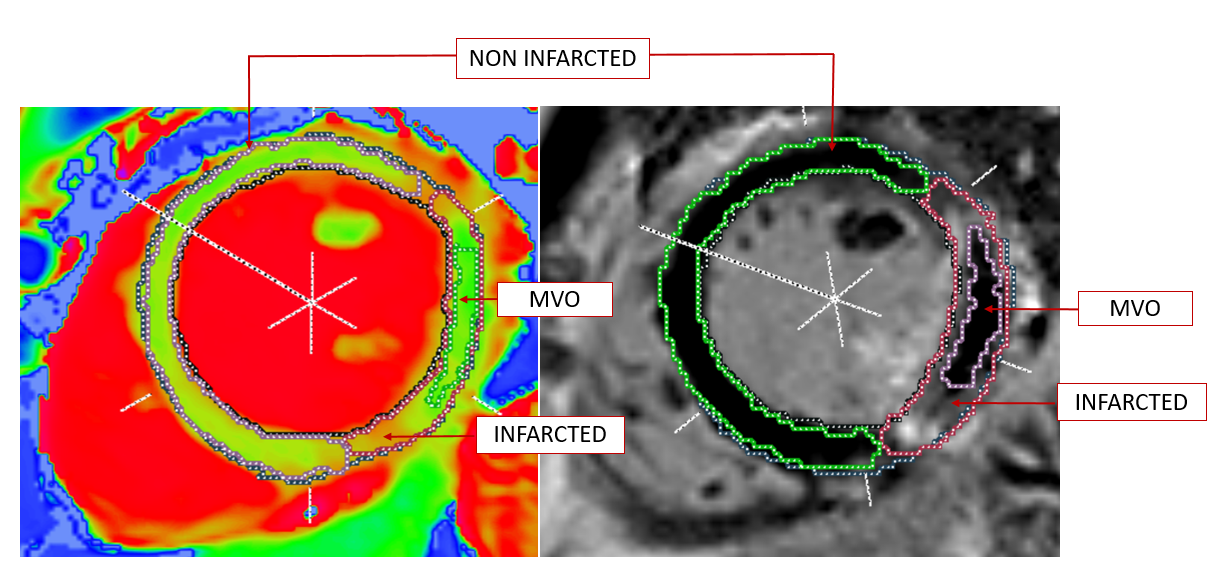

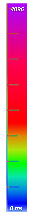

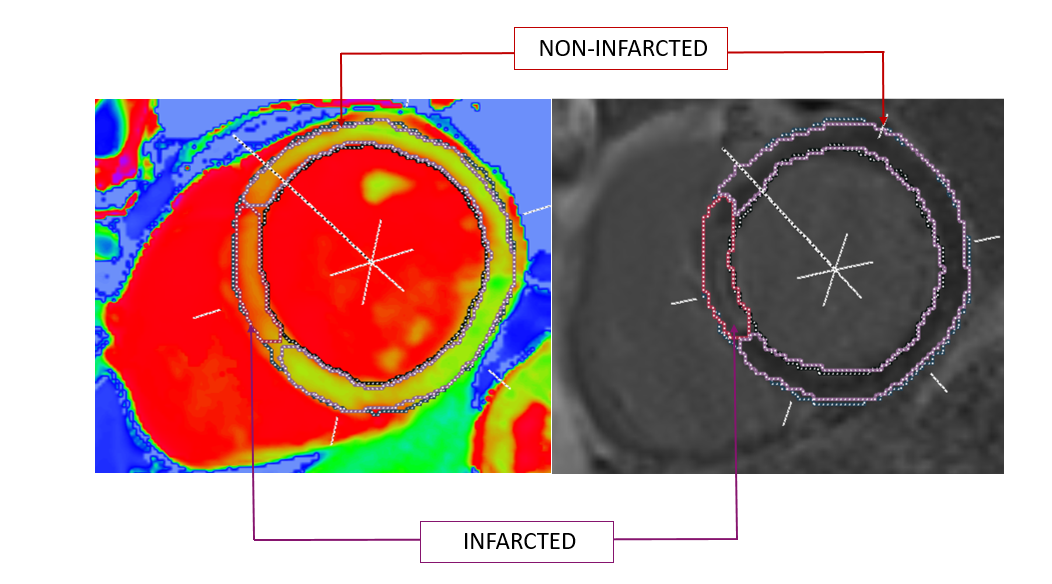

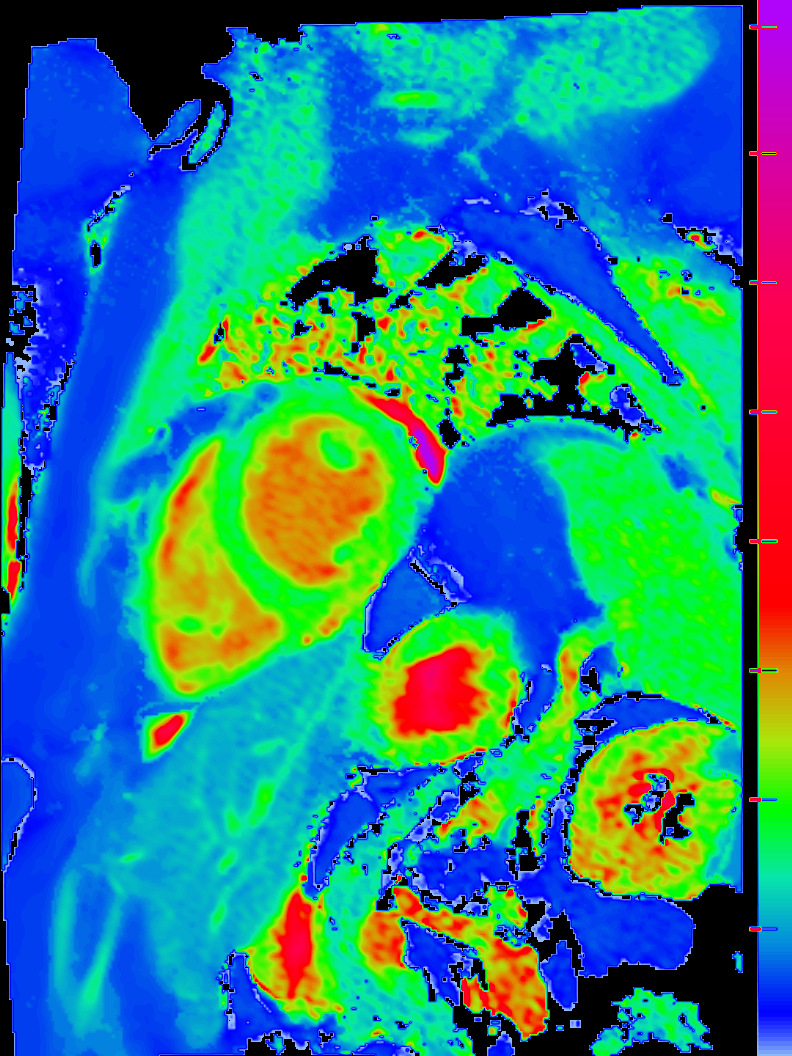


**4096**

**0 ms**

**Supplementary Figures 1-3. Deriving T1 values in infarcted, non-infarcted and remote myocardium.**


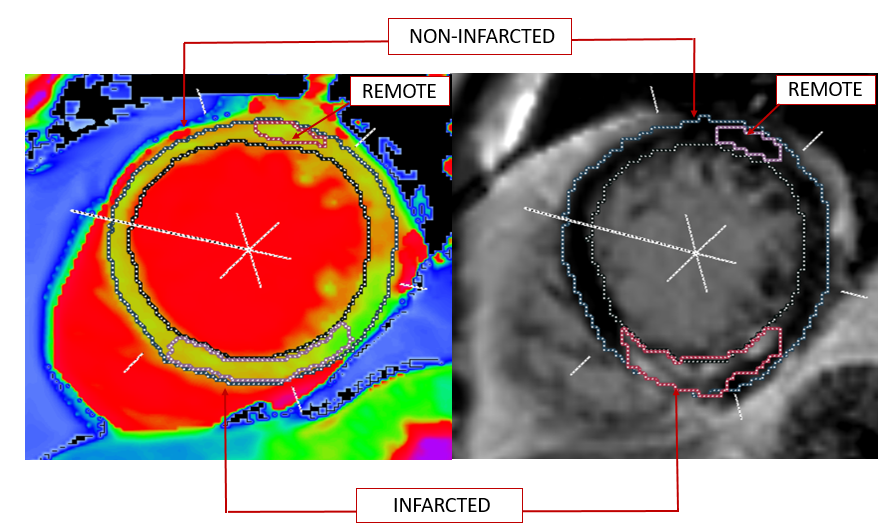

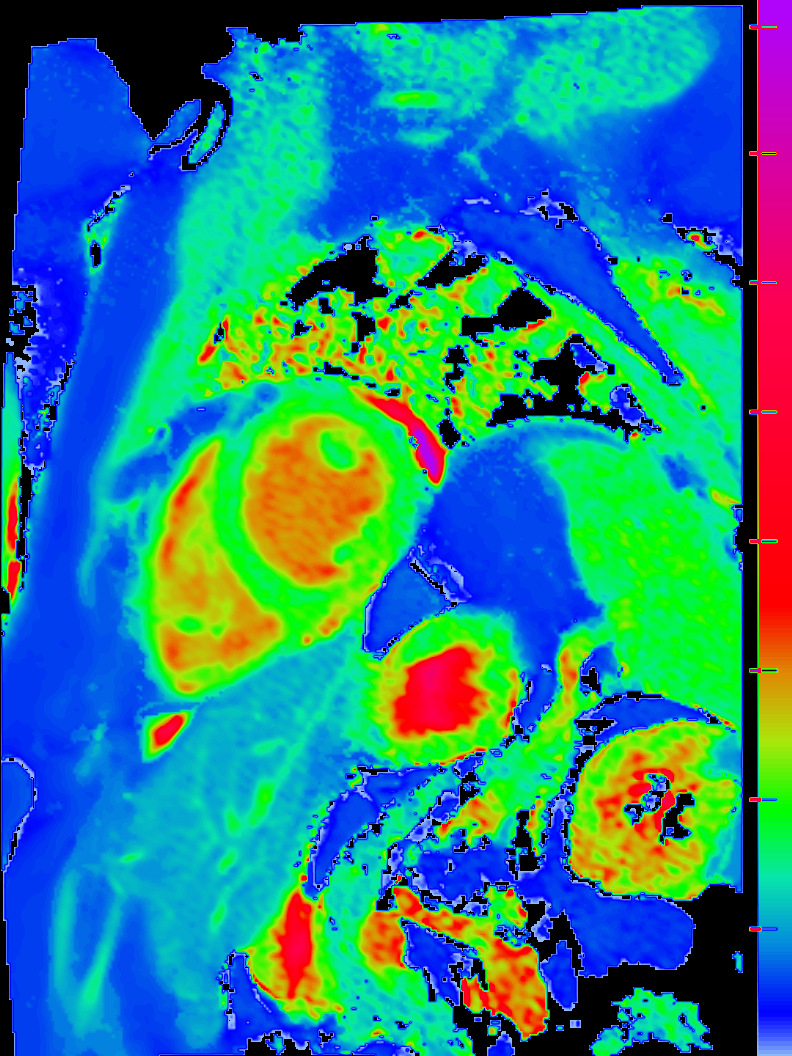


**4096**

**0 ms**

T1-maps and LGE images were obtained at the same slice position in the scanner and compared side-by side to identify the non-infarcted and infarcted myocardial segments on each T1-map. Infarct T1 was derived by averaging the T1 values in all the pixels corresponding to regions with myocardial infarction (including MVO if present). Similarly, the mean non-infarct T1 was calculated by averaging the values all T1 pixels in the entire non-infarcted region. Remote T1 was obtained by drawing a myocardial ROI 180^o^ away from the acutely infarcted segments when possible in regions with no LGE and normal wall motion.

Figure 1 shows a case with MVO and ‘lower infarct T1’. Infarct T1 was relatively lower (<1300ms) in patients with large MVO (>1.55% of entire myocardium). In this case MVO represented 8.94% of the entire myocardium. Mean T1 in the entire infarcted area was low at 1242 ms whilst T1 in the entire MVO area was much lower 1166ms. Figure 2 shows high non-infarct T1 (1330ms). Figure 3 shows high remote T1 (1249 ms).

**SUPPLEMETARY RESULTS**

**Supplementary Table 2 – Multivariate Cox-regression analysis of CMR variables**

| **Model 1:** | | |
| --- | --- | --- |
| **Variable** | **Hazard Ratio (HR) (CI)** | **p-value** |
| Acute LVEF (per 1% increase) | 0.948 (0.890-1.010) | 0.099 |
| Acute infarct size (per 1% increase) | 0.983 (0.938-1.030) | 0.471 |
| **MVO presence** | **3.803 (1.050-13.769)** | **0.042** |
| **Model 2:** | | |
| **Variable** | **HR (CI)** | **p-value** |
| Acute LVEF (per 1% increase) | 0.971 (0.909-1.039) | 0.395 |
| Acute infarct size (per 1% increase) | 0.977 (0.931-1.050) | 0.347 |
| **MVO presence** | **4.295 (1.182-15.607)** | **0.027** |
| **Non-infarct T1 (per 1 ms)** | **1.013 (1.000-1.025)** | **0.042** |
| **Model 3:** | | |
| **Variable** | **HR (CI)** | **p-value** |
| Acute LVEF (per 1% increase) | 1.001 (0.931-1.076) | 0.984 |
| Acute infarct size (per 1% increase) | 0.977 (0.932-1.023) | 0.317 |
| **MVO presence** | **3.747 (1.073-13.0847)** | **0.038** |
| **Non-infarct T1 (per 1 ms)** | **1.025 (1.010-1.040)** | **0.001** |
| **Infarct T1 (per 1 ms)** | **0.986 (0.979-0.994)** | **0.001** |
| **Model 5:** | | |
| **Variable** | **HR (CI)** | **p-value** |
| Acute infarct size (per 1% increase) | 0.978 (0.934-1.025) | 0.353 |
| **Acute LVEF ≤40%** | **3.802 (1.357-10.654)** | **0.011** |
| **MVO presence** | **3.834 (1.052-13.979)** | **0.042** |
| **Model 6:** | | |
| **Variable** | **HR (CI)** | **p-value** |
| Acute infarct size >17% | 1.314 (0.292-5.911) | 0.722 |
| MVO presence | 2.521 (0.711-8.938) | 0.152 |
| **Acute LVEF ≤40%** | **2.852 (1.093-7.442)** | **0.032** |
| **Model 7:** | | |
| **Variable** | **HR (CI)** | **p-value** |
| Acute LVEF **≤**40% | 2.134 (0.742-6.487) | 0.181 |
| Acute infarct size (per 1% increase) | 0.962 (0.916-1.010) | 0.122 |
| **MVO presence** | **4.173 (1.073-13.0847)** | **0.029** |
| **Non-infarct T1 (ms)** | **1.022 (1.008-1.037)** | **0.002** |
| **Infarct T1 (ms)** | **0.987 (0.979-0.995)** | **0.001** |
| **Model 8:** | | |
| **Variable** | **HR (CI)** | **p-value** |
| Acute LVEF<40% | 1.482 (0.538-4.083) | 0.447 |
| MVO presence | 2.470 (0.799-7.630) | 0.116 |
| **Non-infarct T1 (ms)** | **1.020 (1.005-1.034)** | **0.005** |
| **Infarct T1 (ms)** | **0.988 (0.981-0.995)** | **0.001** |
| **Model 9:** | | |
| **Variable** | **HR (CI)** | **p-value** |
| Acute infarct size >17% | 0.910 (0.190-4.361) | 0.907 |
| MVO presence | 2.096 (0.567-7.750) | 0.267 |
| Acute LVEF ≤40% | 2.389 (0.866-6.591) | 0.093 |
| **Non-infarct T1 >1250ms** | **3.651 (1.162-11.471)** | **0.027** |
| **Infarct T1 <1300ms** | **5.988 (2.071-17.310)** | **0.001** |
| **Model 10:** | | |
| **Variable** | **HR (CI)** | **p-value** |
| Acute infarct size (per 1% increase) | 0.962 (0.916-1.010) | 0.118 |
| MVO presence | 3.350 (0.925-12.138) | 0.066 |
| **Acute LVEF ≤40%** | **3.328 (1.160-9.546)** | **0.025** |
| **Non-infarct T1 >1250ms** | **4.361 (1.381-13.776)** | **0.012** |
| **Infarct T1 <1300ms** | **6.251 (2.142-18.237)** | **0.001** |
| **Model 11:** | | |
| **Variable** | **HR (CI)** | **p-value** |
| MVO presence | 2.013 (0.646-6.273) | 0.228 |
| Acute LVEF ≤40% | 2.347 (0.899-6.127) | 0.081 |
| **Non-infarct T1 >1250ms** | **3.643 (1.169-11.357)** | **0.026** |
| **Infarct T1 <1300ms** | **5.982 (2.077-17.227)** | **0.001** |
| **Model 12:** | | |
| **Variable** | **HR (CI)** | **p-value** |
| Acute LVEF (per 1% increase) | 0.977 (0.912-1.047) | 0.507 |
| Acute infarct size (per 1% increase) | 0.974 (0.929-1.022) | 0.289 |
| MVO presence | 3.572 (0.975-13.081) | 0.055 |
| **Remote T1 (per 1 ms)** | **1.015 (1.002-1.028)** | **0.019** |
| **Infarct T1 (per 1 ms)** | **0.990 (0.983-0.997)** | **0.008** |
| **Model 13** | | |
| **Variable** | **HR (CI)** | **p-value** |
| Acute LVEF (per 1% increase) | 0.958 (0.897-1.023) | 0.199 |
| Acute infarct size (per 1% increase) | 0.981 (0.935-1.029) | 0.981 |
| MVO presence | 3.099 (0.869-11.050) | 0.081 |
| Remote T1 >1250ms | 1.996 (0.639-6.231) | 0.234 |
| **Infarct T1 <1300ms** | **3.560 (1.332-9.516)** | **0.011** |

Non step-wise multivariate Cox-regression analysis results. Abbreviation as per Table 1.

**Supplementary Table 3 – Multivariate backward step-wise Cox-regression analysis of clinical factors and T1 indices**

| **Model 1** | | | |
| --- | --- | --- | --- |
| Variables entered: Age, past history of MI, ischaemic time, Troponin, infarct T1 and non-infarct T1 | | | |
| **Variables in the equation at the final step of regression** | | | |
| **Variable** | **Hazard Ratio (CI)** | **p-value** |  |
| Age (yrs) | 1.046 (1.004-1.091) | **0.033** |  |
| Ischaemic time per 1 minute | 1.002 (1.000-1.003) | **0.005** |  |
| Troponin (peak) per ng/l | 1.002 (1.000-1.004) | **0.020** |  |
| Non-infarct T1 per 1ms increase | 1.027 (1.013-1.041) | **<0.001** |  |
| Infarct T1 per 1ms increase | 0.988 (0.980-0.996) | **0.003** |  |

Step-wise multivariate Cox-regression analysis results. Abbreviation as per Table 1.

**Supplementary Table 4 – Multivariate backward step-wise Cox-regression analysis of clinical factors, conventional CMR indices and T1 indices**

| **Model 2** | | | |
| --- | --- | --- | --- |
| Variables entered: Age, past history of MI, ischaemic time, Troponin, infarct T1 and non-infarct T1, LVEF ≤40%, Infarct size >17% and MVO. | | | |
| **Variables in the equation at the final step of regression** | | | |
| **Variable** | **Hazard Ratio (CI)** | **p-value** |  |
| Age (yrs) | 1.050 (1.006-1.096) | **0.024** |  |
| Ischaemic time per 1 minute | 1.002 (1.000-1.002) | **0.008** |  |
| Troponin (peak) per ng/l | 1.002 (1.000-1.004) | **0.022** |  |
| Non-infarct T1 per 1ms increase | 1.027 (1.013-1.040) | **<0.001** |  |
| Infarct T1 per 1ms increase | 0.987 (0.980-0.995) | **0.001** |  |

Step-wise multivariate Cox-regression analysis results. Abbreviation as per Table 1.

**Supplementary Table 5. Comparison of the ability of models containing conventional clinical and conventional CMR indices with and without novel T1 biomarkers to predict MACE**

| **Model** | **Predictors** | **C-statistic** | **Integrated Brier score** | **Comparison of models** | | **Net Reclassification Index (NRI)** | | **Integrated discrimination Improvement Index (IDI)** | |
| --- | --- | --- | --- | --- | --- | --- | --- | --- | --- |
|  |  |  |  |  | |  | |  | |
|  |  |  |  | 𝛘**^2^ difference** | **p** | **index** | **p** | **index** | **p** |
| A | Previous MI, Ischaemic time, troponin peak | 0.67 ± 0.07 | 0.068 |  | | | | | |
| B | Previous MI, ischaemic time, troponin peak, MVO presence, LVEF <40% | 0.71 ± 0.07 | 0.063 | Model A vs B | | | | | |
|  |  |  |  | 7.78 | 0.02 | 30% (CI -9.1-56.8%) | ns | 5.6% (CI -1.6-19.9%) | ns |
| C | Previous MI, ischaemic time, troponin peak, MVO presence, LVEF <40%, non-infarct T1, infarct T1 | 0.77 ± 0.06 | 0.055 | Model A vs C | | | | | |
|  |  |  |  | 19.03 | <0.001 | 54% (CI 0.6-71.3%) | 0.047 | 18.5% (CI 4-35.6%) | 0.013 |
|  |  |  |  | Model B vs C | | | | | |
|  |  |  |  | 11.25 | 0.004 | 26.4% (CI -3.2-60.1%) | 0.086 | 12.9% (CI 1.5-24.8%) | 0.02 |

Comparison of the ability of models with conventional clinical risk factors (A) with conventional CMR indices added (B) and model with T1 indices added (C) to predict clinical outcomes. Abbreviations as per Table 2.

**Supplementary Table 6 – Relationship between peak blood monocyte and neutrophil counts and CRP levels and T1 indices and infarct size acutely after STEMI.**

|  | **Peak monocyte count (10^9^/L)** | **Peak neutrophil count (10^9^/L)** | **Peak CRP**  **(mg/L)** |
| --- | --- | --- | --- |
|  | Correlation (p value) | | |
| **Area of injured tissue** | | | |
| Infarct size measured on LGE (%) | **0.353 (p<0.001)** | **0.271 (p=0.001)** | **0.303 (p<0.001)** |
| Area-at-risk measured on T1 mapping (%) | **0.261 (p<0.001)** | **0.226 (p=0.001)** | **0.195 (p=0.020)** |
| Fraction of myocardium with high T1>1250ms (%) | **0.158 (p=0.024)** | **0.189 (p=0.017)** | **0.157 (p=0.046)** |
| **T1 values within tissue** | | | |
| Infarct T1(ms) | 0.012 (p=0.875) | -0.138 (p=0.102) | -0.033 (p=0.685) |
| Non-infarct T1(ms) | 0.059 (p=0.406) | 0.079 (p=0.328) | 0.092 (p=0.246) |
| Remote T1(ms) | 0.117 (p=0.096) | **0.166 (p=0.038)** | 0.139 (p=0.079) |

Blood results were obtained from serial blood tests performed in the first 48 hours after PPCI for clinical purposes and the peak values were used in this analysis. Spearman (rho) and Pearson (r) correlations were performed as appropriate depending on the distribution of data. Abbreviations as per Table 2.

**Supplementary Tables 7 and 8 - Multicollinearity analysis of variables used in the Cox regression analysis**

| **Variable** | **Variation Inflation Factor (VIF)** |
| --- | --- |
| Left ventricular ejection fraction (LVEF) | 1.64 |
| Infarct size | 1.94 |
| MVO presence | 1.55 |
| Non-infarct T1 (ms) | 1.54 |
| Infarct T1 (ms) | 1.29 |
| **Mean VIF** | **1.59** |

| **Variable** | **Variation Inflation Factor (VIF)** |
| --- | --- |
| Left ventricular ejection fraction (LVEF) <40% | 1.29 |
| Infarct size >17% | 1.33 |
| MVO presence | 1.26 |
| Non-infarct T1 >1250ms | 1.19 |
| Infarct T1 <1300ms | 1.05 |
| **Mean VIF** | **1.22** |

Multicollinearity of variables in the two models was assessed with Variation Inflation Factor (VIF). VIF <5 is considered to be reflect low level of multicollinearity, whilst VIF 5-10 and >10 indicate moderate and high levels of multicollinearity respectively (5,6). Abbreviations as per Table 2.

**Supplementary references**

1. Hann E, Popescu IA, Zhang Q, Gonzales RA, Barutçu A, Neubauer S, et al. Deep neural network ensemble for on-the-fly quality control-driven segmentation of cardiac MRI T1 mapping. Medical Image Analysis. 2021 Jul 1;71:102029.

2. Carapella V, Puchta H, Lukaschuk E, Marini C, Werys K, Neubauer S, et al. Standardized image post-processing of cardiovascular magnetic resonance T1-mapping reduces variability and improves accuracy and consistency in myocardial tissue characterization. International Journal of Cardiology. 2020 Jan 1;298:128–34.

3. Liu D, Borlotti A, Viliani D, Jerosch-Herold M, Alkhalil M, De Maria GL, et al. CMR Native T1 Mapping Allows Differentiation of Reversible Versus Irreversible Myocardial Damage in ST-Segment-Elevation Myocardial Infarction: An OxAMI Study (Oxford Acute Myocardial Infarction). Circ Cardiovasc Imaging. 2017 Aug;10(8):e005986.

4. Zhang Q, Hann E, Werys K, Wu C, Popescu I, Lukaschuk E, et al. Deep learning with attention supervision for automated motion artefact detection in quality control of cardiac T1-mapping. Artificial Intelligence in Medicine. 2020 Nov 1;110:101955.

5. Arezoo B, Midi H. Robust Estimations as a Remedy for Multicollinearity Caused by Multiple High Leverage Points. Journal of Mathematics and Statistics. 2009 Apr 1;5.

6. Stephanie. Variance Inflation Factor [Internet]. Statistics How To. 2015 [cited 2022 May 9]. Available from: https://www.statisticshowto.com/variance-inflation-factor/
